# A Prospective Study of Clinical Presentation, Radiological Findings, and Treatment Outcomes in Patients With Idiopathic Granulomatous Mastitis at a Single Center

**DOI:** 10.7759/cureus.106103

**Published:** 2026-03-30

**Authors:** Jyotsna Oak, Mandar Nandkarni, Bijal Kulkarni, Shefali Sardar

**Affiliations:** 1 Consultant Rheumatologist, Kokilaben Dhirubhai Ambani Hospital and Medical Research Institute, Mumbai, IND; 2 Breast Oncology, Kokilaben Dhirubhai Ambani Hospital and Medical Research Institute, Mumbai, IND; 3 Pathology, Kokilaben Dhirubhai Ambani Hospital and Research Institute, Mumbai, IND; 4 Radiology, Kokilaben Dhirubhai Ambani Hospital and Medical Research Institute, Mumbai, IND

**Keywords:** cystic neutrophilic granulomatous mastitis, hydroxychloroquine, idiopathic granulomatous mastitis, methotrexate, mri breast

## Abstract

Background: Idiopathic granulomatous mastitis (IGM) is a benign, inflammatory breast disease that clinically and radiologically mimics malignancy and infection, posing diagnostic and therapeutic challenges - particularly in tuberculosis (TB)-endemic settings. Evidence on standardized, medical-first strategies in Indian cohorts remains limited.

Objective: To describe the clinical presentation, radiological features, histopathology, and treatment outcomes of IGM managed with corticosteroids, methotrexate (MTX), and hydroxychloroquine (HCQ) at a single center.

Methods: In this prospective cohort (October 2016-June 2025), consecutive patients with core biopsy-confirmed IGM were enrolled after exclusion of infection (including TB polymerase chain reaction (PCR)/culture), autoimmune diseases, and systemic granulomatous disorders. Clinical, laboratory, and multimodality imaging (ultrasonography (USG) and mammography for all; MRI selectively) were recorded. Patients received tapering oral corticosteroids (0.5 mg/kg/day) and upfront MTX (10-15 mg/week); HCQ was added in selected cases. Follow-up included monthly, then three‑monthly assessments with serial labs and imaging. Outcomes were analyzed descriptively.

Results: We identified 110 women (mean age 37.7 ± 7.4 years). Common presentations were breast swelling (41.8%, n=46), palpable lump (30.9%, n=34), pain (22.7%, n=25), and discharge (8.2%, n=9). Imaging typically showed ill‑defined, heterogeneous hypoechoic masses with frequent abscesses, sinus tracts, and reactive axillary nodes; Breast Imaging Reporting and Data System (BI‑RADS) categories were 2 (n=15), 3 (n=29), and 4A-4C (n=66). Histopathology most often demonstrated IGM (48.6%, n=54) and cystic neutrophilic granulomatous mastitis (CNGM; 23.9%, n=26). All patients received corticosteroids plus MTX; most did not require surgery. By 12 months, 76% (n=76) showed clinical improvement on MTX monotherapy; among 100 patients with 12‑month data, 38% (n=38) had no evidence of disease, 56% (n=56) continued treatment, 7% (n=7) were lost to follow‑up, and 9% (n=9) had no improvement. At 18 months, overall, recurrence was documented in nine patients; no clinically significant adverse events were recorded. Median treatment duration was 18-24 months.

Conclusions: In this Indian single‑center prospective cohort, a medical‑first strategy using corticosteroids with early MTX achieved high rates of improvement, low surgical utilization, and acceptable recurrence. Multimodality imaging often necessitated biopsy due to frequent BI‑RADS 4 assignments. These findings support conservative, immunomodulator‑based management and underscore the need for standardized protocols and multicenter prospective validation.

## Introduction

Idiopathic granulomatous mastitis (IGM) is a rare, benign inflammatory breast disease of uncertain etiology, first described by Kessler and Wolloch in 1972 [1]. While its exact cause remains unclear, it is hypothesized to result from autoimmune mechanisms, specifically a granulomatous inflammatory response to ductal epithelial damage, potentially triggered by secretory leakage or an underlying infection [2,3]. IGM primarily affects young women of childbearing age and typically presents with painful breast masses, erythema, abscesses, and skin changes-features that clinically and radiologically mimic inflammatory breast carcinoma, or infectious mastitis [1-4]. Diagnosis is largely one of exclusion and requires histopathological confirmation after ruling out infectious, autoimmune, and systemic causes [2,3]. In tuberculosis (TB)-endemic regions such as India, IGM is frequently presumed to be TB in origin [5,6]. The disease’s unpredictable course, high recurrence rates and physical and psychological burden underscore the need for effective, evidence-based treatment strategies.

Management options for IGM range from surgical excision to pharmacologic interventions. Empirical anti-tubercular therapy and broad-spectrum antibiotics (e.g., amoxicillin-clavulanate, doxycycline) are frequently used despite limited microbiological confirmation. Corticosteroids, including oral, subcutaneous, topical, and intralesional forms, are commonly employed for their anti-inflammatory effects, though long-term use is constrained by adverse effects and relapse risk [7,8]. Methotrexate (MTX), an immunosuppressant, has demonstrated promise as a steroid-sparing agent with favorable outcomes, while hydroxychloroquine (HCQ), owing to its immunomodulatory properties, has gained attention as adjunctive therapy, particularly in recurrent or steroid-resistant cases. Surgery is typically reserved for patients who do not respond to conservative treatment. Although often successful, surgical management is associated with prolonged healing, delayed reconstruction and disfigurement [9,10].

Despite the growing use of pharmacologic therapies, there is no consensus on the optimal treatment strategy for IGM [11], and real-world data on therapeutic outcomes in Indian patients remain limited. Given the heterogeneity in diagnostic approaches and treatment responses, a clearer understanding of therapeutic effectiveness is essential for clinical decision making. This study aimed to describe the clinical, radiological, histopathological, and treatment-related outcomes of IGM in a single-center cohort, with treatment outcomes reported descriptively rather than as a formal assessment of therapeutic efficacy.

## Materials and methods

Study design and setting

Consecutive patients with histopathologically confirmed IGM diagnosed between October 2016 to June 2025 were included in this study. Study was conducted at Kokilaben Dhirubhai Ambani Hospital and Medical Research Institute. Clinical presentation, personal and obstetric history, and findings on breast examination were recorded.

Diagnostic workup, treatment, and follow-up

Baseline investigations included complete blood count (CBC) and routine blood biochemistry, including blood glucose levels. Definitive diagnosis was established in all patients by 14-gauge core needle biopsy. Biopsy specimens were divided into two portions: one was fixed overnight in 10% formalin for histopathological examination, and the other was placed in a sterile container for TB polymerase chain reaction (PCR) testing and culture (TB PCR reports available within 24 hours). Mantoux testing, chest X-ray, and evaluation for autoimmune diseases - including rheumatoid factor (RF) and antinuclear antibody (ANA) - were performed in all patients.

Although IGM lacks pathognomonic imaging features, serial imaging was used to monitor lesion progression, assess treatment response, and detect recurrence or new disease foci. Imaging modalities included high-resolution ultrasonography (USG), mammography, and breast MRI for diagnostic evaluation and lesion characterization. USG and mammography were performed in all patients, whereas breast MRI was reserved for selected cases with inconclusive sonomammographic findings [11].

Inclusion criterion required biopsy-proven granulomatous inflammation confined to the breast lobules, composed of polymorphonuclear inflammatory cells and/or epithelioid and giant cells without caseation necrosis. No pathogenic organisms, including acid-fast bacilli, were identified on Periodic Acid-Schiff (PAS), or Gram staining. MTX and corticosteroid therapy was initiated only after infectious causes were excluded.

Patients with culture-proven active infection were excluded. All patients were counseled regarding contraception throughout the treatment period and for three months following discontinuation of MTX.

Patients were initially followed up monthly and subsequently at three-month intervals. At each visit, clinical assessment included measurement of breast lump size and body temperature, along with laboratory monitoring of liver function tests (LFTs), renal function tests (RFTs), and CBCs. All patients were followed for a minimum of 12 months to assess for disease recurrence. In patients receiving MTX, CBC and LFTs were monitored regularly, and drug doses were adjusted accordingly. All cases were independently reviewed by both a pathologist and a rheumatologist.

Data collection

Data collected included age, sex, clinical history, laterality of breast involvement, lesion size, findings from mammography and sonography (where available), histopathological subtype, type and duration of treatment outcomes and recurrence. All combinations of medical and surgical management were documented.

Ethical considerations

Institutional Review Board approval was obtained from the ethics committee (ISEB Code: C-3/32/2015).

Statistical analysis

Descriptive statistical analyses were performed to summarize the demographic, clinical, imaging, and treatment-related characteristics of the identified cases with IGM diagnosis. Continuous variables, such as age and time to diagnosis, were expressed as means with SDs (mean ± SD). Imaging characteristics were summarized to identify common radiological patterns using frequency distributions and qualitative categorization. Lesion type, location, size, and associated Breast Imaging Reporting and Data System (BI‑RADS) classifications were described, according to the American College of Radiology (ACR) BI‑RADS® Atlas (2013) [12]. Key imaging descriptors - such as hypoechoic lesions, abscesses, cystic components, heterogeneous echotexture, and lymphadenopathy - were analyzed to delineate recurring radiological features of IGM.

We enrolled 110 consecutive biopsy‑proven cases, representing 96.5% of the calculated requirement, which is acceptable for descriptive studies, especially in rare conditions such as IGM. 

## Results

A total of 110 cases of IGM were identified, all were female patients. The mean age at presentation was 37.7 ± 7.4 years (range: 24-59 years) (Table 1). The average duration from initial presentation to diagnosis was six months.

**Table 1:** Frequency distribution of patient age. The majority of patients were between 30-40 years of age, indicating a predominance of cases in this reproductive-age group (n=110)

Age	No. of patients
20-30	11
31-40	67
41-50	23
More than 50	09

Clinical presentation

The left and right breasts were involved with approximately equal frequency. Bilateral breast involvement was observed in a small proportion of cases.

Breast Swelling

**Figure 1:**
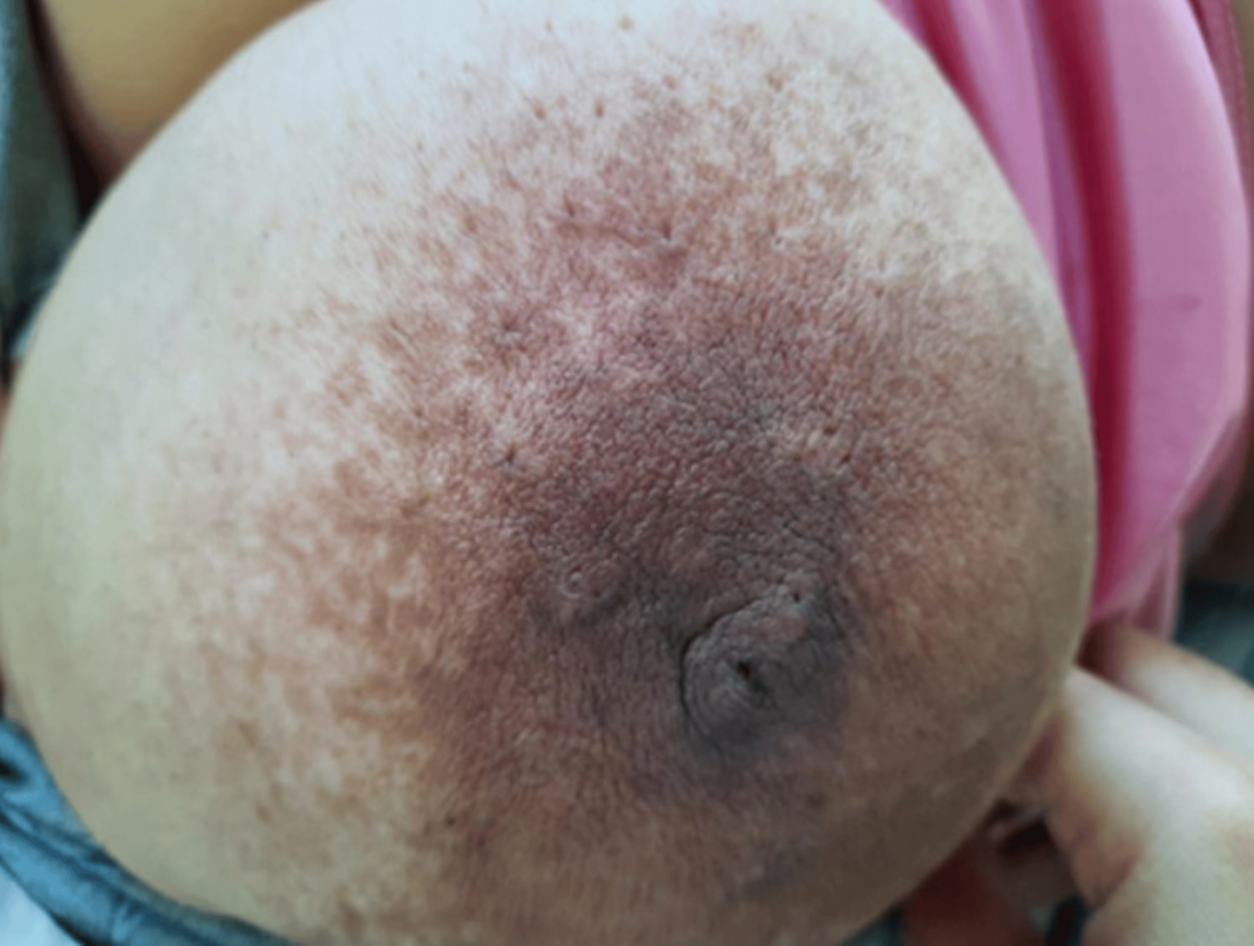
Breast swelling with erythema

The most frequent presenting symptom was breast swelling, reported in about 42% of patients (n=46), with variable laterality and symptom duration ranging from two to four months in some patients (Figure 1).

Breast Lump

A palpable breast lump was reported in over 30% of the cases (n=34), often described as firm or hard, with some cases associated with increasing size over time and persisting for three-four months. A substantial proportion of patients reported recurrent swelling or abscess, suggesting a chronic or relapsing course of IGM.

Breast Pain and Redness

Pain was commonly reported in about 23% of cases (n=25), often in conjunction with swelling or lump formation. Descriptions of painful nodules, throbbing sensations, erythema, and tightness were noted. In about 20% of cases, systemic symptoms such as fever and weakness were also present, reflecting a more active inflammatory phase.

Nipple Discharge and Pus

Discharge, including purulent or serous fluid, was reported in about 8% of cases (n=9). This was occasionally associated with visible sinus tracts, particularly in chronic or recurrent cases, further supporting the suppurative nature of IGM in some presentations.

Atypical Presentations

While the predominant symptomatology aligned with classical IGM, a few patients reported less typical findings such as intermittent itching (n=1), cyclical mastalgia (n=1), or breast tightness with small nodules (n=1) with no associated symptoms.

History of Recurrence and Chronicity

A few patients (n=11) presented with a documented history of IGM or recurrent mastitis/abscess, with symptom onset dating back several months to years before coming to our center.

Surgical History

Of the 110 patients, 15 patients had some form of surgical procedure -primarily incision and drainage for abscesses (n = 10), followed by surgical excision for abscesses (n = 5).

Patient imaging characteristics

Among the 110 cases, imaging findings were predominantly derived from USG examinations, followed by mammography, with occasional references to MRI, and are reported in a standardized format specifying lesion location (by clock position), size, echogenicity, and BI-RADS classification (Table 2).

**Table 2:** Analysis of sonomammography findings with the respective number of patients, with maximum patients having irregular heterogenous hypoechoic lesions (61%) (n=110)

Sonomammography findings	No. of patients	Percentage of cases
Irregular heterogenous hypoechoic lesions	67	61%
Axillary lymph nodes	29	26.30%
Abscess formation	7	6.36%
Periductal mastitis	7	6.36%

**Figure 2:**
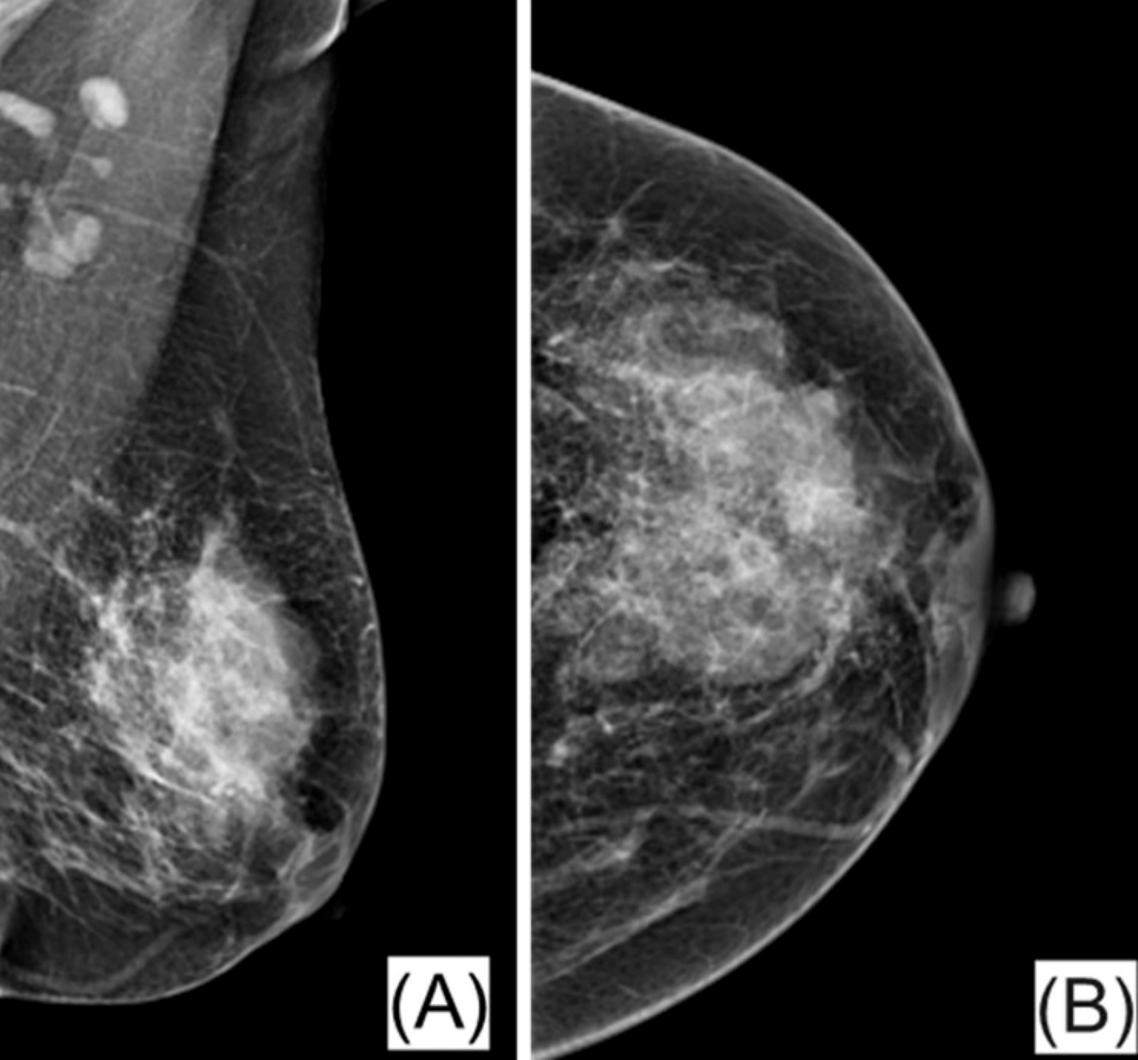
Sonomammography: (A) The irregular heterogeneous mass lesion with slightly indistinct margin noted at the site of palpable concern in the left breast superiorly (from 11-2 o'clock) could be suggestive of granulomatous mastitis and a core biopsy is recommended. Few ducts distended with hypoechoic contents are noted extending to the retroareolar region; (B) An enlarged left axillary lymph node with preserved hilar fat and another smaller one with displaced hilar fat are noted. (BI-RADS – 4B) BI-RADS: Breast Imaging Reporting and Data System

The imaging reports revealed a wide spectrum of morphological patterns, anatomical distributions, and BI-RADS categories, consistent with the heterogeneous clinical behavior of IGM (Figure 2).

Lesion Morphology and Echotexture

The majority of lesions (n=84) were described as ill-defined, heterogeneous, and predominantly hypoechoic masses across multiple quadrants of both breasts varying in size from small (e.g., 0.6×0.2 cm at 1 o'clock) to large (e.g.,6.7×3.0×3.0 cm at 7-9 o'clock). Several lesions demonstrated solid-cystic architecture, increased vascularity, and posterior acoustic enhancement, suggestive of inflammatory infiltrates. In selected cases, lesions were nodular and/ or demonstrated tubular extensions or irregular contours, raising suspicion for malignancy.

Laterality and Quadrant Involvement

Lesions were distributed bilaterally, with involvement of both the left and right breasts. The most frequently affected regions were the upper outer quadrants (1 o’clock -3 o’clock), lower inner quadrants (9-6 o’clock), and retroareolar and periareolar zones

Multifocal and multicentric involvement was commonly observed, with lesion dimensions ranging from 0.6×0.2 cm to 7×7×4.8 cm. Some lesions extended across quadrants or toward the nipple and intradermal layer, consistent with inflammatory infiltration and sinus formation.

Abscess Formation and Fluid Collections

Well-defined and complex fluid collections were reported in over one-third of cases, with typical features including dense internal echoes, mobile debris, and thick walls - reflecting chronicity or organization of inflammatory debris. Abscesses were often located in the retroareolar region, extending medially or toward the skin. Abscesses of varying size ranging from small (e.g., 0.9×0.7cm) to large (e.g., 7×7×4.8cm) were noted. These findings reinforce the suppurative nature of IGM, which can evolve from granulomatous inflammation to necrosis and abscess formation.

Sinus Tracts and Fistulous Communications

Subcutaneous sinus tracts were visualized in multiple patients, frequently originating from the periareolar or subareolar regions, with tract lengths ranging up to 3 cm and discharging sinus tracts at 3, 6, and 9 o'clock positions.

Lymphadenopathy

Enlarged axillary lymph nodes were documented in both axillae. Lymph nodes were described as enlarged or with cortical thickening suggesting reactive changes secondary to the IGM inflammatory process. In most cases, these were interpreted as reactive or granulomatous, though a subset of lesions was associated with BI-RADS 4A-4C classifications, necessitating biopsy to rule out malignancy.

Ductal and Cystic Changes

Cystic lesions and dilated ducts are also reported, though less frequently. Dilated ducts (e.g., 0.43 cm dilated duct) with echogenic contents were noted, particularly in the retroareolar regions, suggesting periductal mastitis or duct ectasia. In addition, small cysts, complex cysts, and fibroadenomas, typically benign, were reported in BI-RADS 2 cases.

MRI features

MRI studies revealed non-mass enhancement with conglomerates of tubular hypoechoic structures, large segments involvement (e.g., 12-3 o'clock), multifocal patterns and soft tissue nodules, especially in the upper outer quadrants. The MRI modality aided in better delineation of disease extent and was particularly useful in multifocal or recurrent presentations. These findings were crucial in cases with inconclusive ultrasound and aided in lesion mapping and surgical planning.

BI-RADS classification

The radiological classification according to the BI-RADS varied widely. The BI-RADS classifications assigned to the lesions provide insight into their perceived malignancy risk and guide clinical management. The report includes a range of BI-RADS categories (Table 3).

**Table 3:** Frequency distribution of BI-RADS score. The majority of patients showed BI-RADS 4 (n=110). BI-RADS: Breast Imaging Reporting and Data System

BI-RADS Score	No. of patients	Percentage of cases
BI-RADS 2	15	13.63%
BI-RADS 3	29	26.36%
BI-RADS 4	66	60%

BI-RADS 2 (Benign)

15 cases: Well-defined small cysts and hypoechoic lesions, fibroadenomas. These lesions are considered benign with no further workup required, though their presence in the context of IGM may reflect coexisting benign pathology.

BI-RADS 3 (Probably Benign)

29 cases: Organized collections, mild asymmetry. These lesions indicate a low risk of malignancy but require short-term follow-up. These lesions often represent inflammatory or granulomatous changes that warrant monitoring.

BI-RADS 4A-4C (Suspicious for Malignancy)

66 cases: Lesions with ill-defined borders and heterogeneous echotexture, i.e., irregular, hypoechoic, and vascular lesions with skin involvement or axillary lymphadenopathy. These lesions, such as the large heterogeneous mass or the complex solid-cystic mass necessitate biopsy to rule out malignancy, as IGM can mimic breast carcinoma on imaging.

BI-RADS 5 (Highly Suggestive of Malignancy)

No cases were reported indicating limited instances of high-certainty malignancy.

Histopathological findings

**Figure 3:**
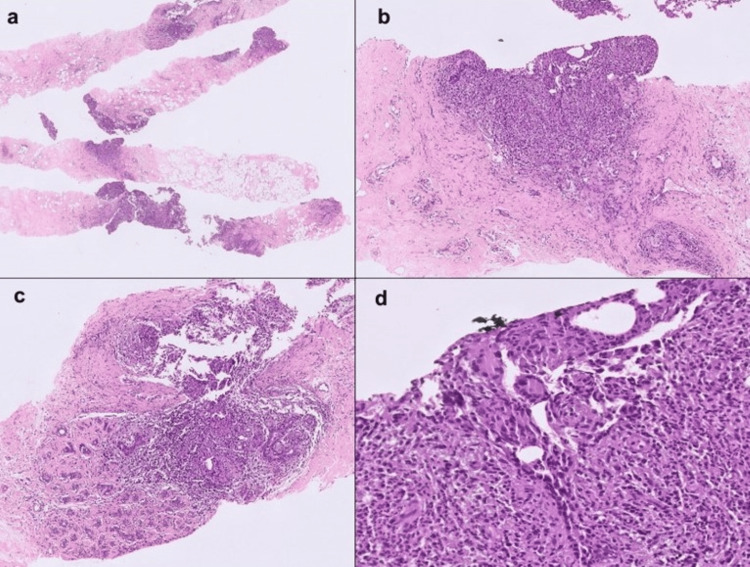
(A) Breast cores showing inflammation around the ducts and lobules sparing the intervening stroma - H&E, 2x. (B) Periductal granulomatous inflammation with giant cells - H&E, 10x. (C) Perilobular inflammation - H&E, 10x. (D) Granulomas involving the ducts with giant cells - H&E, 40x. H&E: Hematoxylin and eosin

Histopathological reports were available for the majority of patients, revealing a diverse spectrum of findings with characteristic inflammatory patterns, underscoring the heterogeneity of IGM presentations (Figure 3).

IGM was the most frequently reported histopathological diagnosis, accounting for approximately 48.6% of cases (Table 4).

**Table 4:** Showing maximum cases of IGM (43.6%), followed by CNGM (23.9%).(n=110) IGM: Idiopathic granulomatous mastitis; CNGM: Cystic neutrophilic granulomatous mastitis

Histopathology findings	No. of patients	Percentage of cases
CNGM	26	23.9%
Lobulocentric mastitis	16	14.7%
IGM	54	48.6%
Breast abscess	7	6.4%
Periductal mastitis	7	6.4%

Cystic neutrophilic granulomatous (CNGM) constituted 23.9% of cases and was characterized by epithelioid histiocytic granulomas, often accompanied by small cystic spaces, neutrophilic infiltrates, and variable degrees of necrosis. This predominance highlights CNGM as a distinct morphological variant within the IGM spectrum, often associated with suppurative features and lobulocentric involvement.

Treatment outcomes

All patients were treated with a short course of corticosteroid for two to three months in tapering doses. Upfront treatment with MTX was given to all patients for 9 to 12 months. Corticosteroids were given at a dose of 0.5 mg/kg daily orally which was gradually tapered according to patient response. MTX dosing was started at 10 mg/week and increased to 15 mg/week given per orally and doses were modified based on clinical response.

Within the first 12 months of treatment, 76% patients noted improvement of their disease with escalating doses of MTX as monotherapy. At 18 months, 80% had disease improvement. Median duration of treatment was 18-24 months. At 12 months, 38 of 100 patients demonstrated no evidence of disease and remained disease-free at follow up on average of two years, 56 had ongoing treatment, seven were lost to follow up, and nine failed to improve on treatment. Resolution was seen in all cases - a few patients had recurrences evaluated at 18 months. There were no side effects seen in any of the patients.

HCQ was added for most patients for remission and they responded well to treatment. Most patients did not require surgical intervention to excise the affected breast tissue.

Outcome

To date, resolution was observed in all patients except nine who experienced recurrence. Notably, some had a history of cyclical mastalgia, while the others presented with purulent discharge and breast tightness with small nodules, respectively.

## Discussion

This prospective study provides critical insights into the clinical, radiological, histopathological, and treatment outcomes in a cohort of 110 Indian patients diagnosed with IGM. Consistent with global patterns, all 110 patients in our study were women of reproductive age, with a mean age of 37.7 ± 7.4 years, slightly on the higher side of age range. The findings highlight the heterogeneous nature of IGM, its diagnostic complexity, and the effectiveness of pharmacologic management, particularly with corticosteroids and MTX, in achieving favorable outcomes with low recurrence rates. These results contribute to the growing body of literature on IGM, particularly in TB-endemic regions like India, where distinguishing IGM from infectious etiologies such as TB mastitis remains a critical diagnostic challenge.

The clinical presentation of IGM in this cohort aligns with prior studies, with breast swelling, palpable lumps, erythema and pain being the most common symptoms [2,3]. The predominance of one-sided involvement (99%) and rare bilateral cases (1%) are consistent with patterns reported in the literature, though the reasons for this laterality remain unclear [3]. Notably, atypical presentations such as intermittent itching and cyclical mastalgia, observed in a small subset of patients, underscore the diverse clinical spectrum of IGM and the need for heightened clinical suspicion to avoid misdiagnosis with inflammatory breast carcinoma or infectious mastitis [2]. Importantly, nearly 11% of patients had a documented history of recurrence, and (8%) had previously undergone incision and drainage procedures, reinforcing the chronic and relapsing nature of IGM and the importance of long-term follow-up.

Radiologically, our findings reaffirm IGM’s diagnostic complexity. Ultrasonographic features were variable, ranging from hypoechoic, ill-defined, heterogeneous lesions to multifocal and multicentric lesions, abscesses, sinus tracts and axillary lymphadenopathy. These findings often mimicked breast malignancies, with 66 patients assigned BI-RADS 4A-4C classifications. Such imaging ambiguity highlights the diagnostic challenge of distinguishing IGM from breast carcinoma and the need of histopathological confirmation, often necessitating biopsy, to prevent misdiagnosis and overtreatment [13-19]. MRI, though used in a limited number of cases, proved valuable in delineating disease extent, particularly in multifocal or recurrent cases, with recommendations for its use in complex or inconclusive presentations [20-24]. The presence of BI-RADS 2 and 3 lesions in a subset of cases suggests coexisting benign pathology, which may reflect the inflammatory milieu of IGM. These radiological insights emphasize the importance of integrating imaging with histopathology to achieve an accurate diagnosis.

Histopathologically, IGM emerged as the most common. CNGM, characterized by lobulocentric granulomas with neutrophilic debris and microabscesses, has been increasingly recognized as a distinct and possibly immune-mediated form of IGM [18]. There were several reports of lobulocentric or ductocentric granulomatous mastitis, indicating the granulomatous inflammation was centered around breast lobules or ducts. These were sometimes non-necrotizing in nature, a key feature that helps differentiate IGM from infectious granulomatous diseases such as TB. Lobulocentric and ductocentric patterns suggest anatomical specificity, while chronic and non-necrotizing forms indicate variability in inflammatory duration and nature. Rare findings, such as foreign body reactions, highlight potential etiological diversity. The absence of acid-fast bacilli and caseous necrosis in all cases supports the non-infectious etiology of IGM, a critical diagnostic criterion in regions with high TB prevalence. Many specimens displayed chronic inflammatory infiltrates, including lymphocytes, plasma cells, and histiocytes, with Langhans-type multinucleated giant cells observed in a subset, further supporting a granulomatous process.

A smaller group of biopsies noted foreign body-type giant cells and chronic suppurative inflammation, especially in specimens obtained from abscess walls, suggesting an ongoing or organizing abscess phase of the disease.

Notably, a subset of samples was reported as acute on chronic mastitis, consistent with IGM’s relapsing-remitting nature. Overall, these pathological findings affirm the heterogeneity of IGM. The disease exhibits a spectrum ranging from acute inflammation to well-formed granulomas, often centered around breast lobules or ducts, and typically lacking caseous necrosis or identifiable infectious organisms.

The predominance of CNGM in our study lends support to emerging evidence that neutrophilic-driven inflammation may be a hallmark of IGM, with implications for targeted immunomodulatory therapy.

IGM often resolves spontaneously within 6-24 months, with nearly half of cases requiring no active intervention. For small, mildly symptomatic lesions, supportive care with anti-inflammatory agents, including MTX, and patient reassurance may suffice [11,15,17,18]. However, cases with marked inflammation or purulent discharge may warrant pharmacological management. Therapeutically at our clinic, all patients received systemic corticosteroids and upfront MTX, with the majority showing complete resolution. Only three patients experienced recurrence, suggesting that early initiation of MTX may reduce relapse rates and serve as an effective steroid-sparing strategy. These findings align with previous studies that advocate for MTX in the management of steroid-refractory or relapsing IGM [25-28]. Our use of HCQ in selected cases also reflects a growing interest in its immunomodulatory role, although larger prospective studies are needed to substantiate its efficacy [29].

Surgical intervention was limited to incision and drainage or excision in selected patients with abscess formation, consistent with current recommendations to avoid surgery unless absolutely necessary due to the risk of poor wound healing and cosmetic deformity [5]. This shift toward pharmacologic management aligns with recent trends favoring conservative approaches to minimize complications such as prolonged healing and delayed reconstruction. This conservative approach is further supported by our data, which demonstrate favorable outcomes with medical management alone in the vast majority of patients.

Clinical and diagnostic implications

The imaging findings underscore the complex and heterogeneous nature of IGM. Frequent involvement of the retroareolar and periareolar regions, along with inflammatory features such as mastitis and edema, aligns with the known pathophysiology of granulomatous inflammation centered around lactiferous ducts. The presence of sinus tracts and fistulae is particularly suggestive of IGM and helps distinguish it from infectious mastitis or breast carcinoma.

The predominance of lobulocentric and ductocentric granulomatous inflammation necessitates histopathological confirmation via core biopsy or excision, given the clinical and radiological overlap with malignancy and infection. The BI-RADS categorization, particularly BI-RADS 4, reflects the diagnostic uncertainty surrounding IGM and explains the need for biopsy to rule out malignancy. The presence of bilateral lesions and axillary lymphadenopathy may further complicate diagnosis, raising concern for systemic or multifocal pathology.

The detection of abscesses and mixed inflammatory infiltrates-comprising lymphocytes, plasma cells, and neutrophils-warrants comprehensive microbiological and histopathological evaluation to exclude infectious etiologies. The chronic and relapsing course of IGM, evidenced by chronic inflammatory changes, highlights the need for long-term follow-up and individualized therapeutic strategies, including anti-inflammatory or immunosuppressive agents.

Collectively, these findings carry important clinical implications. Firstly, IGM should be a key consideration in the differential diagnosis of inflammatory breast disease, particularly in TB-endemic regions where granulomatous mastitis may be misattributed to TB. Second, accurate diagnosis and management require a multidisciplinary approach involving radiologists, pathologists, and rheumatologists. Third, early initiation of immunosuppressive therapy-especially MTX-may reduce recurrence and minimize the need for surgical intervention.

Strengths and limitations

This study’s strengths include its relatively large sample size (n=110) for a rare histologically confirmed condition; the comprehensive analysis of clinical, radiological, and histopathological data, and the focus on a TB-endemic population, which adds a unique perspective to the global literature on IGM and adds to the growing body of evidence supporting immunomodulator therapy over surgical approaches. All our patients underwent core biopsy and TB PCR to rule out TB in a high-risk setting such as India. The standardized treatment protocol and low recurrence rate provide valuable insights into effective management strategies. 

Patients were followed-up for a minimum of six months to up to two years after complete resolution of IGM.

However, limitations include the selection bias inherent to single-center experiences. Future prospective studies should incorporate molecular diagnostics to further elucidate IGM’s etiology and optimize treatment protocols.

Future directions

The findings of this study underscore the need for standardized diagnostic and treatment guidelines for IGM, particularly in regions with high infectious disease burdens. Prospective, multicenter studies are warranted to validate the efficacy of corticosteroid-MTX regimens and explore the role of adjunctive therapies such as HCQ. Additionally, research into the immunological and molecular mechanisms underlying IGM could identify novel therapeutic targets, potentially reducing reliance on corticosteroids and their associated adverse effects. The integration of advanced imaging techniques, such as elastography and contrast-enhanced MRI, may further refine diagnostic accuracy and guide treatment planning.

## Conclusions

This study provides critical insights into the clinical, radiological, and therapeutic aspects of IGM in an Indian cohort. Our findings reinforce the utility of histopathology in diagnosis and support early initiation of corticosteroids and MTX as an effective, conservative management strategy. Future prospective studies are needed to refine diagnostic and treatment algorithms, evaluate long-term outcomes, and explore the role of adjunctive therapies such as HCQ.
